# “Imprisoned” in pain: analyzing personal experiences of phantom pain

**DOI:** 10.1007/s11019-014-9555-z

**Published:** 2014-03-20

**Authors:** Finn Nortvedt, Gunn Engelsrud

**Affiliations:** 1 Faculty of Health Science, Institute of Nursing, Oslo University College of Applied Sciences, Pb. 4 St. Olavs Plass., 0130 Oslo, Norway; 2 Department of Physical Education, Norwegian School of Sport Science, PB 4041, Ullevål Stadion, 0806 Oslo, Norway

**Keywords:** Pain, Body, Phantom pain, Metaphors, Phenomenology

## Abstract

This article explores the phenomenon of “phantom pain.” The analysis is based on personal experiences elicited from individuals who have lost a limb or live with a paralyzed body part. Our study reveals that the ways in which these individuals express their pain experience is an integral aspect of that experience. The material consists of interviews undertaken with men who are living with phantom pain resulting from a traumatic injury. The phenomenological analysis is inspired by Zahavi (J Conscious Stud 8(5–7):151–167, 2001) and Merleau-Ponty (Phenomenology of perception. Routledge and Kegan Paul, London, 1962/2000). On a descriptive level the metaphors these patients invoke to describe their condition reveal immense suffering, such as a feeling of being invaded by insects or of their skin being scorched and stripped from their body. Such metaphors express a dimension of experience concerning the self that is in pain and others whom the sufferer relates to through this pain, as well as the agony that this pain inflicts in the world of lived experience. This pain has had a profound impact on their lives and altered their relationship with self (body), others and the world. Their phantom pain has become a reminder of their formerly intact and functioning body; they describe the contrast between their past and present body as an ambiguous and disturbing experience. We conclude that these sensitive and personalized experiences of phantom pain illuminates how acts of expression—spoken pain—constitute a fundamental dimension of a first-person perspective which contribute to the field of knowledge about “phantom pain”.

## Introduction

Phantom pain has attracted a great deal of attention in research literature, as well as in philosophical treatises. This is a subject that concerns some of the most significant and crucial aspects of the interrelationship between mind and body, and can be regarded as a prototype of the mind–body dilemma (Devor 2004). Among the more significant contributions to the literature have been those of pain scientists (Melzack and Wall 1982/1996; Melzack 1993, 1999, 2005; Devor 1997) and contemporary philosophers such as Merleau-Ponty (1962/2000). Descartes’ Principles of Philosophy (1644/1983) remains the classic text on the subject. This paper was inspired by our own interest in phantom pain, not only as a philosophical issue, but as a lived experience.

Phantom pain can be defined as “[p]ain referring to a missing part of the body or to the paralyzed part of the body after a total spinal lesion” (Nortvedt 2006, p. 13). A century and a half ago, the American neurologist Silas Weir Mitchell used the word “phantom” to label the experience of feeling/sensing a “missing” limb, which he encountered while attending to a multitude of amputees during the American civil war. He characterized this experience as an “unseen ghost of the lost part” (Mitchell 1871). Patients often describe their phantom pain as a burning, cramping or itching sensation (Nikolajsen and Jenssen 2006). Some people experience phantom pain after a spinal cord injury; it can take the form of a cramping pain below a total lesion of the spinal cord. Researchers have also recorded instances of phantom pain following a tooth extraction or removal of a breast or a body organ such as the bladder, uterus or genitalia. (Marbach and Raphael 2000; Ramachandran and Hirstein 1998; Ramachandran and McGeoch 2007).

Roughly 60–80 percent of patients experience phantom pain after losing a limb or body part. There seems to be no correlation between its occurrence and the patient’s gender or age (Nikolajsen and Jensen 2006). The pain can last a lifetime. In a study of several thousand soldiers who had lost a limb, Sherman (1997) found that more than 70 percent continued to experience phantom pain as long as 25 years after the amputation.

Phantom pain is characterized by a sensation that the pain is emanating from a limb that is still intact—and sometimes that is still functioning. These sensations can be quite frequent; in some cases, even more frequent than the pain itself. After the loss of a limb, between 90 and 98 percent of patients experience a phantom vividly (Ramachandran and Hirstein 1998). They may report feeling a phantom arm swinging when they are walking, or a phantom knee bending when they are sitting down on a chair (Melzack 1992). Some studies have found that the phantoms are more intense and enduring following traumatic limb loss or amputation to alleviate a pre-existing painful pathology (*ibid*). After a spinal cord injury, the phantom sensation often emerges immediately, and usually fades away after a while. Sometimes, patients feel their paralyzed legs moving freely and uncontrollably in the air.

Some patients report that the phantom body part appears fixed in a strange and very painful position (Melzack 1992). One patient cited felt as if his phantom arm extended straight forward from his shoulder; to avoid banging it when walking through doorways, he always turned sideways while crossing a threshold (*ibid* 1992). Another patient could not lie on his back while sleeping because he felt as if the phantom arm was bent in a strange position behind his back. Other patients have claimed that they can generate voluntary movements in their phantom limb (Ramachandran and Hirstein 1998).

The capacity of people to experience pain in a lost or paralyzed body part poses interesting questions for the health sciences and other disciplines. This paper explores ways in which individuals with phantom pain articulate and understand their pain and their situation.

## Theories of phantom pain

Ronald Melzack (1993, 1999, 2005) has posited one recent phantom pain theory. Drawing on the immense amount of new information being generated in cognitive neuroscience, he argues that future research on phantom experience and phantom pain should focus on a much deeper understanding of brain functioning. Though there is still much to learn about peripheral mechanisms (e.g., the precise functions of the spinal cord and midbrain descending control systems), he observes, the brain beyond the midbrain merits even greater exploration. He argues that this need is evident from the perspective of our empirical knowledge of phantom pain and the phenomenon of phantom sensations, as experienced among tetra- and paraplegics as well as among limb amputees (*ibid*). As Melzack puts it,

There is no better way to enter this exciting world than to consider phantom limbs and phantom bodies: “The body self” that is still present in experience even when input from that part of the body is gone. (Melzack 1993, p. 621)

Drawing on his observations and empirical facts, he posits a theory of a “neuromatrix”: a broad, distributed neural network that plays a major role in our cognitive and emotional perception and our awareness of pain. Melzack notes that even children born with congenital limb deficiency can have a phantom perception and experience pain in a limb they have never had, and argues that this is evidence of a distributed neural representation of the body that is in part genetically determined (Melzack et al. 1997; Ramachandran and Hirstein 1998).

In characterizing the neuromatrix as “the template of the whole,” providing a characteristic and neural pattern for the entire body, Melzack sharply contradicts the earlier specificity theory, which proposed that experiential qualities like pain were inherent in peripheral nerve fibers (Melzack and Wall 1965). It could be argued, however, that when Melzack (1993) proposed subsequently that the brain generates the body’s experience, he might have been underestimating the role of embodiment in pain perception and experience. It is possible, for example, that the whole body is a phantom, and Melzack’s neuromatrix is a type of “brain mythology” that views the brain as a physical annex separate from the body. Fuchs makes this argument against Melzack’s theory in a cogent critique (2002, p. 321):

It (the brain) perceives, learns, hypothesizes and commands as if it were a living being of its own. Neuronal circuits are attributed intentional and meaningful behaviour, as if they were some kind of homunculi. This is only the counterpart of reductionism; reducing personal consciousness to sub-personal mechanisms results in personalizing these mechanisms.

We concur that theories that describe phantom pain as a purely neural event and localized perception in the mind have distinct limitations. These theories don’t *explain* the phenomenon; they just *describe* it as a purely neural event in a conscious brain. But phantom pain is not experienced in the *mind* as an isolated perception; it is experienced in the *body*, by a living human being.

Dealing with phantom pain is dealing with an aspect one of the most difficult problems in modern philosophy; the nature of the mind and the relationship with body and mind. The French philosopher Merleau-Ponty (1962/2000) reframed the relationship between subject and object, self and world, primarily through a radically different perspective on the lived body. He argued that the significance of the body, or the body-subject, is too often underestimated, through a tendency to consider it simply as an object that a transcendent mind orders to perform a variety of functions. In opposition to this, he proposed an embodied inherence in the world that is more fundamental than our reflective capacities. Our perceiving mind, according to Merleau-Ponty (1962/2000), is an incarnated body; the mind is inseparable from our embodied and physical nature. Viewing ourselves through the lens of being-in-the-world as embodied subjects could explain a phenomenon like the phantom limb, in which the body is experienced as an irreducible whole even when parts of it are removed. From this perspective, phantom pain might be an experience that evolves through the interrelationship between a person’s current and past experiences, and not primarily as an activity in a cerebral matrix of conscious sensation, as posited by Melzack (1993, 1999, 2005).

Merleau-Ponty specifically discusses the ambiguity of the phantom limb experience, in which the body is experienced as an irreducible entirety even when parts of it are removed. He argues that the experience of the phantom limb is a manifestation of an inborn complex, but also posits that the limb can come into existence through the individual’s experience of situations that become internalized through memory (Merleau-Ponty 1962/2000 pp. 84–85). We will return to this ambiguity later when discussing our empirical material. In response to current theories and our interest in this particular type of pain, we developed the following research questions (Nortvedt 2006, p. 11):

- How do individuals describe their experiences with pain in a lost body part or a paralyzed body?
- In what ways does the phantom pain express itself as an embodied experience?
- What is the relationship between the phantom pain and the lost or paralyzed body part?
- In what ways is the phantom pain related to individuals’ experience of the world and other people?

## Methods and materials

To examine these research questions, we recruited eight men as informants. Our recruitment of informants followed accepted and ordinary research ethical standards, and the National Committee for Research Ethics (REK) approved the study. We selected our group of eight in close collaboration with nurses at a rehabilitation unit. All of these nurses had extensive experience with pain problems; they had known and observed all eight of our informants for a long period. It should be noted that none of these informants were women. At the time of our study, no women with phantom pain were being treated at the unit. This was not surprising. Most people who suffer a traumatic injury that results in severe phantom pain are men, and (thankfully) it is a relatively rare phenomenon. Our informants ranged in age from 20 to 50. In each case, their severe and persistent phantom pain began after a traumatic limb amputation or a total lesion of the spinal cord. To gather empirical material from these informants, the first author conducted qualitative in-depth interviews with them and engaged in participant observation.

The first author conducted research at the rehabilitation unit over the course of 6 months. During this period he conducted participant observations (Fangen 2004) and followed the patients during a variety of daily activities, such as training and social interactions. Each observational period concluded with an in-depth interview in which the informant was asked to describe his pain and how it affected his life. The interviews lasted between 45 and 75 min. The primary focus was on how the informant described his experiences with his past and present body and how he spoke of the pain in the context of his past and present experience.

The researcher began each interview by asking the informant to talk about his pain and describe it in his own words. In every one of these interviews, the informant responded by talking freely and with great openness about his pain. They seemed to regard the opportunity to discuss their pain and their situation with someone who was extremely attentive as both meaningful and comforting. It is noteworthy that all of the informants discussed their pain using language that was rich and metaphorical. The first author also interviewed three health care professionals from the unit—a nurse, a physiotherapist and an occupational therapist—concerning their experiences with patients who experienced phantom pain. All three were experienced professionals who had worked with chronic pain patients in the unit for many years.

The first author transcribed all of the interviews verbatim. In analyzing them, we applied what Kvale (1997) describes as a dialectical relationship between reading and reflecting on texts. Our analysis was also informed by the similar strategy suggested by Miller and Crabtree (1999), which they call immersion/crystallization. In this approach, interpreters couple their readings of the transcribed texts with ongoing reflections. In other words, they try to create a dialectical structure in their interpretation and presentation of the material.

## Analysis

The following section contains our analysis of the material, which shows how patients express their pain through their body and language, and describes how these expressions are related to phantom pain.

### Pain engenders sensation of the body

In the interviews, the patients described their experiences of limbs or body parts as if they still belonged to an intact body. They talked about their feeling of being painfully embodied and experienced lost body parts or paralyzed bodies parts in distinct ways *through* their pain—as body parts with pain. When they felt pain, they could feel the intactness of the lost limb or the paralyzed body part as vividly as if it were still there. The pain seemed engender the sensation of the body and became a re-actualization of their formerly whole and functioning body. The body returned through the pain; one could say that pain reminded the body of itself. While experiencing episodes of phantom pain, a tetraplegic man with a complete lesion of the spinal cord described feeling the lower part of his lost foot:

Even though I am totally paralyzed from the neck down, I still have a feeling of being in contact with every part of my body. Because of the painful itching I know where my legs are, and through the pain I can feel my knees and toes as if they were there.

Similarly, a young man who had become a paraplegic after a car accident described being “woken up” by the pain, as if the toes on his foot were growing into each other:

I was lying in bed here at the hospital and feeling really bad. All day I felt as if the nail on one toe was growing into the adjacent toe. My mother was visiting, and I asked her if she could take a look and see what was happening. She couldn’t see anything wrong. Another day, I woke up with the same feeling; the pain had kept me up most of the night.

A young patient who felt phantom pain in his right foot after a motorbike accident described the way it sometimes shot down his leg, as if the leg were still intact. He made noises of shots and electricity to illustrate this—*thaa, thaa* ….zzz, zzz., At one point, he described the phenomenon this way:

The pain can sometimes shoots out, *thaa*,… down in the foot like it was still there, and I can feel pain in my “knee” and down in my “toes,” as if they were still intact. So the pain can be localized in a strange way.

The phantom pain seems to anchor the body, which means that it provides “bodyness,” or that the pain *has* body. In this manner, the pain becomes a reminder of the former whole and functioning body, and the body returns through the pain. One might say, then, that their phantom pain makes these individuals feel embodied and reminds the body of itself.

### The language of phantom pain

Our informants expressed the phenomenon of phantom pain through metaphorical language. The men both expressed and characterized the metaphors as they talked about how the pain relates to the body, creating visual and precise descriptions. Their metaphors were extremely violent and brutal, and their stories revealed immense suffering—of feelings such as being stabbed by knives or burnt by a fire in which their skin was being ripped off. One spoke of a sensation that he was being invaded by insects, not only crawling all over his skin, but through his veins:

— and it itches! But it’s very difficult to explain. It’s as if I am lying in a nest of insects, and they’re constantly crawling not only outside but inside my body.

A patient whose right arm and leg had been amputated after a motorbike accident described the excruciating pain in his arm:

It’s as if the skin of my arm has been ripped off; salt is being poured on it and then it’s thrust into fire. I also sometimes feel as if the fingers on my amputated hand are moving uncontrollably, which is both extremely painful and embarrassing.

Through the invocation of metaphors, these patients provide an inter-subjective perspective that conveys a common dimension of everyday life that could be a significant method for conveying and communicating their pain to others. It may also be an important strategy for coping with the pain in the course of their daily life. The act of relating stories and experiences of phantom pain can improve the patients’ situation, and at the same time enhance health care workers’ understanding of what it is like to live with these types of pain.

### “Painful” doubt

Our informants also described their struggles to comprehend their phantom pain. The way they posed questions reflecting a variety of assumptions about their situation: what is mind? What is body? What is my situation? As we have seen, these questions are not restricted to the patients. They are related to one of the primary questions in pain research and philosophy in general: How is it possible to experience pain in a body part that no longer exists? In the light of the long dualistic tradition in Western science and philosophy, one can understand why these patients seem to doubt the reality of their own experience. Not surprisingly, they appear to find this doubt disturbing. From a research point of view, it also reflects the dilemmas that confront these patients in their desperate efforts to restore their embodied self as a unified and comprehensible entity. The way in which they talk about their phantom pain and how it relates to their body clearly indicates that defining their pain as either exclusively physical or exclusively psychic is neither logical nor valid. From the perspective of our informants, pain is always a lived, embodied experience. One of them expressed this dilemma and his doubts about the pain he felt in this fashion:

I don’t really have pain, do I? Because I’m paralyzed. I mean, if you’ve lost your arm, you can’t feel pain in an arm you don’t have any more, right? So in a way, it has to be something psychic, something you imagine.

The patients convey their doubt in the language of “daily life,” expressed in terms like “just my imagination” and “this isn’t real.” However, the material we gathered shows that phantom pain may be intensified by this constant reminder of the existence of a lost limb, where pain has become the dominant representation of an earlier and functional body. As the quotations above indicate, this can be a disturbing experience that confines individuals with lost or paralyzed limbs psychically and shuts them off from the world. The way in which phantom pain confronts patients with their former existence as individuals with a whole and functioning body has a profound existential dimension. Their body is not their body as it was before. It is their previous body in a distorted form. This may become a haunting and troubling experience.

Some of our informants labelled their pain as a vicious enemy that threatened to ruin their lives. They insisted that they would rather have been disembodied if that would have allowed them to escape the pain. Comments from two of them illustrate this desperation:

If I had been totally paralyzed, cut off from my previous body, [I would be happier.]

What has become dead should be dead.

In the latter sentence, the informant was expressing his unbearable suffering, and the dilemma of having to exist and live with a body part in which the only sensory experience is excruciating pain. Another young man, on the other hand, compared the loss of his leg with the loss of a close friend or relative:

I feel that it can be compared with a feeling of grief, the kind of grief you can experience after the loss of a dear, old friend or family member. But it’s also a reminder. The phantom pain reminds me that my leg is gone forever, so I don’t get any opportunity to forget that. It’s remarkable how easy it is now to notice the normality of having two legs. When I see a football match on TV or other sporting activities, I always think about how I’m shut out of these activities; I can’t engage in them anymore, and that troubles me….

This traumatic experience occupies considerable space in the informant’s life, and creates feelings of marginalization and sadness. He is troubled by the permanent loss of his leg, a loss that he is constantly reminded of by the pain, and suffers from the “double pain” of having his literal pain related to the missing limb compounded by the psychological “pain” of having lost the limb.

### Pain as a threat to life

The experiences related by these patients were also imbued with a profound existential angst. The pain they felt compelled them to confront their former existence as individuals with a “whole and functioning body.” In these situations, remembering one’s former self and previous body inevitably generates a feeling of vulnerability, as well as haunting and troubling experiences. For some of the men, continuing life in such a situation seemed an unbearable prospect. The pain was so devastating and terrible that they spoke of suicide as the last and only option if nothing else brought them sufficient relief. All of our informants described the intensity of their pain and its strange character as a constant and enduring reminder of a former existence. Their perceptions of their own bodies were altered in the strangest ways, and they spoke of being under unbearable physical and psychological strain. One patient described it this way:

There are times when I just want… just want to drive my bed into the water and end it all. If I could have done it by myself I would have. That would have put an end to all this shit.

Another, a man, about 20 years old, spoke of his depression and his thoughts of suicide:

It’s constant, as if I have a big, strange object growing out on my head that I can’t get rid of. It’s always there and it drives me crazy. It does something terrible to me. I can’t concentrate on anything but the pain. If I read a book I can’t concentrate on the text and I don’t remember what I’ve read. I can’t go on living like this.

The ward perceived this patient as “the happy boy”—always smiling and chatting, with a sharp, witty comment for everyone, his fellow patients as well as the nurses and doctors. Yet in the interview he spoke openly about the possibility of ending his life to escape the pain. He had not mentioned these thoughts to anyone other than the interviewer and the health professionals on the ward did not seem to be aware of them. He reflected on this during the interview and explained that no one could possibly comprehend what it’s like to live with such terrible pain. He preferred to be labelled as the “happy boy,” and not trouble anyone else, either other patients or staff on the ward, with his problems. His “happiness” also helped him be sociable with others, which he regarded as an important aspect of his daily life in the institution.

### Enjoying life in spite of pain

The patients’ desire to go on living despite experiencing a pain so strange and intense that it could be threatening to life itself suggests a profound ambiguity. Whether implicitly or explicitly, our informants expressed a belief that things can’t get worse; they have to get better. They showed tremendous courage and a strong will. Some were finding solutions that made their life worth living, after all. Several even suggested that things could be worse. As one patient put it, “Even in this situation, you have to see the possibilities and not the limits.”

Most of our informants exhibited great strength of character. They saw their pain as unavoidable, a reality that they could not escape and had to accept. One of them spoke of his future life this way:

I look forward to the day when I can begin working again. To the day when I can read a book and remember its contents. Right now, the pain is so terrible that I just forget. The pain steals all of my concentration so I don’t remember what I read. I look forward to the day when I have children, when a little girl or boy runs up to me saying, “Hi daddy!” So everything is still exciting in a way. You have to think differently about everything, but the future is still exciting—although I wish it could be free of pain!

This informant exhibited a striking ambivalence. He had a strong will to live and a belief that life can be exciting in spite of his pain, while at the same time he desperately wanted the pain to disappear, or at least become less intense. But life must go on; there seems to be a passion for life itself that is so powerful and demanding that it diminishes misery, and even seemingly unbearable agony. Life itself transcends everything, even excruciating and constant pain!

The search for meaning in a life that is shattered by pain was clearly articulated by one of our informants:

I have an attitude to life that tells me that everything has meaning. People think, why me? Why poor me, why couldn’t this have happened to someone else? But I think that this happened to *me* for a reason, and that the meaning is that I can cope with this, this situation, I can manage to make life go on….

This informant is devoting a great deal of his time to visiting schools, where he warns students about all the dangers in traffic and how exposed you are when riding a motorbike at high speed. He believes that telling other young people about his own accident and situation may teach them something that prevents them from getting into a similar situation. This new purpose provides him with the energy and courage to go on living.

## Further analysis: phenomenology and phantom pain

The empirical material we gathered reveals that phantom pain is an experience that transforms and alters an individual’s perception of the self and his relationship to the world and others. In our further analysis, we have chosen to read the material and structure our discussion from the perspective of Danish philosopher Dan Zahavis’s (2001) triad of the World, Others and the Self. Zahavi, argues that these three concepts (or regions) belong together and reciprocally illuminate one another; they should be understood through their interrelationship.

As already indicated, we discovered that the way our informants talked about others and themselves was intimately connected with their relationship to others and the world. Their pain was not experienced as “internal,” but as relational and bodily—both constant and fluctuating. Based on this finding, we decided to use Merleau-Ponty’s interpretive framework, in which phantom pain is an ambiguous experience that is both here and now and a reminder of the individual’s formerly whole and functioning body.

Zahavi emphasizes that a first-person perspective should not be confused with the classical transcendental and idealistic project of detaching the mind from the world so that its richness and concreteness can be embodied in a pure and wordless subject (2001). According to Zahavi (*ibid*), the subjective does not have priority over the world, and “truth” cannot be found in our interiority. Priority, he argues, belongs to individuals in the world; they know themselves through living in the world. The subjectivity disclosed by its phenomenological reflection is an open world-relationship, embedded and embodied in a social, historical and concrete context.

### Relating to the world and the self

Phantom pain changes an individual’s relationship to the world; it also alters the world’s relationship to the individual. As Merleau-Ponty observes (1962/2000, p. 165),

I can close my eyes, lie down, listen to the blood pulsating in my ears, lose myself in some pleasure or pain, and shut myself up in this anonymous life which subtends my personal one. But precisely because my body can shut itself off from the world, it is also what opens me out upon the world and places me in a situation there. The momentum of existence towards others, towards the future, towards the world can be restored as a river unfreezes.

In this quotation Merleau Ponty describes a situation that is related to the subject’s openness towards worldly existence and the momentum that allows individuals to shut *off* their relationship to the world. But for the men in this study, this possibility of being *shut off* is precarious due to the intensity and character of their phantom pain. This pain torments them to such a degree that it alters their perception of being in the world as embodied persons. For them, being anchored to the body feels like being trapped. Their existence becomes enclosed in their pain-filled body, absorbing all of their attention. Scarry (1985, p. 35) describes the devastating effect:

It is the intense pain that destroys a person’s self and world, a destruction experienced spatially as either the contraction of the universe down to the immediate vicinity of the body or as the body swelling to fill the whole universe.

The pain never seems to give them peace; it follows them throughout the day and into the night. Their stories reveal immense suffering and they see no end to it. The pain becomes so disrupting and devastating that at times some of them talk about ending their lives. At the same time, they do not regard their situation as hopeless or irreversible. This is apparent in the manner in which our informants talked about their hope for the future and their will to live in spite of the pain. They are able to focus not only on their problems, but on other possibilities, particularly how their situation could be worse: “I am not the one who is worst off; others are in even more difficult situations,” is the way they commonly express this. They seem to find a kind of comfort in this type of comparison with others.

Though many of the informants were injured in a motorcycle accident, nearly all of them want to ride a bike again and continue their lives in much the same way as before. This retrospective link to the past and earlier habits expresses their will not to give up, and to resume their earlier lifestyle. One of the most interesting and important tendencies in the material we elicited is the ambiguity expressed in comments concerning their past and current body. Our informants find themselves trapped by their pain and also removed from the entire texture of life. They feel imprisoned, separated from themselves, from life and from others. They describe themselves as both cut off and shut off. At the same time, their pain ties them to both their body and their remembered wholeness. It is possible that this is what motivates some of these men to speak of ending their lives, of wanting to be disembodied: From their perspective, being in the world immerses them in the ambiguity of their relationship to the person they once were and the person they are now.

### Relating to others

The patients in our study expressed a deep feeling of loneliness; of being cut off from sharing a meaningful community with others. Phantom pain is a shattering and devastating experience that others cannot understand and even our informants themselves could not fully comprehend. They spoke of their isolation, an isolation based on their certainty that others, whether health workers or relatives, could not adequately comprehend their situation. Typically, other people relate to the tangible result of the accident and regard the amputation or paralysis as the patient’s main problem. According to our informants, however, it was the invisible pain that was shattering their life. They think of it as “hidden” from others, and express it using metaphors. At the same time, they want to socialize and share in the community of others. They are intent on mixing with others, even though this entails constant, extremely debilitating struggle. They are adamant that they do not want to be a burden to others. They try to behave stoically. Although this stoicism might be a strategy to protect themselves and others from the pain, it also allows them to engage in a certain amount of social life that does not belong to the pain. Their lives seem to be split into two different spheres, as there is little correspondence between how they experience their situation and how they express it to others. This bifurcation can intensify their loneliness.

In his book *Body Silent,* Robert Murphy (1990) describes his life as a quadraplegic. He explains how his life had changed and the ways in which his bonds and connections with others had been altered. In addition, he describes how his paralysis and dysfunction threatened his masculine identity. Here is how he describes the latter difficulty:

Paralytic disability constitutes emasculation of a more direct and total nature. For the male, the weakening and atrophy of the body threaten all the cultural values of masculinity; strength, activeness, speed, virility, stamina and fortitude (pp. 94–95).

In contemporary society, life on the road, speeding along on a motorcycle is a social and cultural symbol of “ideal” masculinity, linked to a life of freedom and independence. The men in this study seemed to struggle hard to protect their masculine identity. This may partly explain their desire to resume their former habits. In Murphy’s terms, the stamina or fortitude that this behavior expresses is motivated by the will to forge a new masculine identity that at least partly recapitulates former habits and behavior.

The way in which these individuals talk about their pain makes them attentive to their embodiment. One could ask: “Is this pain given the position of a “friend” or an “enemy”? Based on interviews with patients with spinal cord injuries, Cole (2004) argues that pain may become a friend, because it allows the individual to stay in contact with his paralyzed body. Though the pain is intense and causes suffering, some of the individuals with spinal cord injuries whom he studied preferred to live with their pain because it reminded them of their former functional body” (Cole, ibid).

The opposite may also be true. The informants in this study did not speak of their pain as a friend that kept them in contact with their former bodies. Rather, they regarded it as an enemy that worsened their imprisonment. As noted earlier, some of them explicitly said that they would rather feel disembodied if that would allow them to escape their pain. We also noted earlier that many of the informants spoke of their struggle to comprehend how the pain could occur in a body part that was either “lost” or paralyzed. They questioned and doubted their own experience, and it could be argued that this doubt reflects the existential dilemmas that people live through when they desperately try to restore their body image as unified and comprehensible.

### Articulating the pain

Words, as previously illustrated in some of the violent and brutal metaphors used by our informants, express the immensity of the pain they experience. They create an impression reminiscent of torture. But in this case, the torture is not inflicted from outside by others; it is their own body that tortures them. Their body has turned against them. Some of our informants explicitly characterized the pain as an enemy they have to fight against. The violent descriptions and metaphors are reminiscent of a war.

In ordinary life, when we experience pain, we relate it to an object and localize it somewhere in our material body. Phantom pain has no connection with a functioning object, a body part. However, those who experience it seem to develop their own object, a metaphor that relates to the body so that the pain becomes understandable. From this perspective, their metaphors can be seen as the pain’s object, making it comprehensible by reconnecting the inner and outer world.

For informants in our study, vocalizing their phantom pain served as a mechanism of embodiment. Their pain did not destroy language, as Scarry (1985, p. 5) proposes when she writes,

Physical pain does not simply resist language but actively destroys it, bringing about an immediate reversion to a state anterior to language, to the sounds and cries a human being makes before language is learned.

Merleau-Ponty’s perspective provides insight into the questions posed by the way our informants use metaphors to express their pain. He argues that there is no understanding without language, without speech. The speaker does not think before speaking, nor even while speaking; his speech *is* his thoughts (*ibid*, p. 180). Thought is accomplished through expression and becomes apparent at its original level of embodiment (Thøgersen 2005).

Merleau-Ponty’s (1962/2000) characterization of the link between speech and thought and the intentionality and expressions of the body are consistent with the ways in which our informants used language to express their pain. Words, he suggested, are located in each individual’s linguistic world, and become a manifestation of intimate being. They represent part of our ability to communicate, because the voice is replete with gestures, and by expressing emotions it has the capacity to affect others. This is particularly important for someone who is immobilized and challenged by a restricted repertoire of gestural language. Articulating the pain through speech can be an important link to the world of others, a way to break the isolation the pain creates. Individuals in pain partly validate its reality by having others listen to their experiences. Our informants used metaphors to shape language into an embodied expression of their pain. For them, as Merleau-Ponty suggests (1962/2000), language became a manifestation of the intimacy and unity between individuals, the world and others inhabiting the same world.

Merleau-Ponty (1962/2000) sees body image as a conclusive way of establishing that our bodies are in the world. In his view, being-in-the-world in a pre-reflexive way is the fulfilment of the relationship between the physical and psychic dimensions of being. The experience of the body as whole is a result of intentionality and of being-in-the world as a body-subject. This experience reveals that the body is related to the world in all its parts and that we exist as embodied persons even if parts of our body have been lost or paralyzed. From this perspective, we experience the body as an irreducible whole rather than as the embodiment of a genetic construct or cerebral neuromatrix as Melzack argues (1993). In other words, our experiences of the body may be a consequence of intentionality and being-in-the-world as embodied subjects. The findings of this study are consistent with this perspective.

Merleau-Ponty further suggests that habituation and past remembrance of our body might play a crucial role when experiences of phantom pain are comprehended and perhaps even explained. As he puts it (1962/2000, p. 91):

When we try to elucidate the phenomenon of the phantom limb by relating it to the body image of the subject, we add to the accepted explanations, in terms of cerebral tracks and recurrent situations, only if the body image, instead of being the residue of habitual cenesthesis, becomes the law of its constitution.

## Conclusion

Eliciting intimate expressions of phantom pain from a group of male informants has enabled us to confirm that it can become a haunting experience that “traps” them in their past bodies. They suffer from constant and enduring pain with an intensity and character that can shatter and destroy their lives. Their pain severely constrains their relationships with others and with their bodies, as well as their ability to exist in the world. At the same time these men experience the pain with an ambiguity in which they struggle to hold on to former habits and at the same time create hope and “new ways” to sustain life, despite their extreme agony. They try to facilitate and “create” new habitual bodies by engaging in new activities, managing to propel their wheelchair by themselves and making plans for the future.

Through the insights these informants shared, we have been able to understand that phantom pain might become a reminder of the former functional body, which has re-emerged in a distorted form. The ambiguity they experience between their past and present body might be a disturbing experience that is difficult to comprehend. At the same time, it suggests that phantom pain has profound existential components. Applying a phenomenological perspective has enabled us to discuss and illuminate how acts of expression—“spoken pain”—constitute a fundamental dimension of experience comprised of the self that is in pain, the others that are encountered through that pain, and the world of the lived experience of that pain.
